# The variant rs77559646 associated with aggressive prostate cancer disrupts *ANO7* mRNA splicing and protein expression

**DOI:** 10.1093/hmg/ddac012

**Published:** 2022-01-19

**Authors:** Gudrun Wahlström, Samuel Heron, Matias Knuuttila, Elina Kaikkonen, Nea Tulonen, Olli Metsälä, Christoffer Löf, Otto Ettala, Peter J Boström, Pekka Taimen, Matti Poutanen, Johanna Schleutker

**Affiliations:** Cancer Research Unit, Institute of Biomedicine, University of Turku, 20520 Turku, Finland; FICAN West Cancer Centre, University of Turku and Turku University Hospital, 20520 Turku, Finland; Cancer Research Unit, Institute of Biomedicine, University of Turku, 20520 Turku, Finland; FICAN West Cancer Centre, University of Turku and Turku University Hospital, 20520 Turku, Finland; Research Centre for Integrative Physiology and Pharmacology, Institute of Biomedicine, University of Turku, 20520 Turku, Finland; FICAN West Cancer Centre, University of Turku and Turku University Hospital, 20520 Turku, Finland; Turku Center for Disease Modeling (TCDM), University of Turku, 20520 Turku, Finland; Cancer Research Unit, Institute of Biomedicine, University of Turku, 20520 Turku, Finland; FICAN West Cancer Centre, University of Turku and Turku University Hospital, 20520 Turku, Finland; Cancer Research Unit, Institute of Biomedicine, University of Turku, 20520 Turku, Finland; FICAN West Cancer Centre, University of Turku and Turku University Hospital, 20520 Turku, Finland; Cancer Research Unit, Institute of Biomedicine, University of Turku, 20520 Turku, Finland; FICAN West Cancer Centre, University of Turku and Turku University Hospital, 20520 Turku, Finland; Cancer Research Unit, Institute of Biomedicine, University of Turku, 20520 Turku, Finland; FICAN West Cancer Centre, University of Turku and Turku University Hospital, 20520 Turku, Finland; Department of Urology, Turku University Hospital, 20520 Turku, Finland; Department of Urology, Turku University Hospital, 20520 Turku, Finland; Cancer Research Unit, Institute of Biomedicine, University of Turku, 20520 Turku, Finland; FICAN West Cancer Centre, University of Turku and Turku University Hospital, 20520 Turku, Finland; Department of Pathology, Turku University Hospital, 20520 Turku, Finland; Research Centre for Integrative Physiology and Pharmacology, Institute of Biomedicine, University of Turku, 20520 Turku, Finland; FICAN West Cancer Centre, University of Turku and Turku University Hospital, 20520 Turku, Finland; Turku Center for Disease Modeling (TCDM), University of Turku, 20520 Turku, Finland; Cancer Research Unit, Institute of Biomedicine, University of Turku, 20520 Turku, Finland; FICAN West Cancer Centre, University of Turku and Turku University Hospital, 20520 Turku, Finland; Department of Medical Genetics, Genomics, Laboratory Division, Turku University Hospital, 20520 Turku, Finland

## Abstract

Prostate cancer is among the most common cancers in men, with a large fraction of the individual risk attributable to heritable factors. A majority of the diagnosed cases does not lead to a lethal disease, and hence biological markers that can distinguish between indolent and fatal forms of the disease are of great importance for guiding treatment decisions. Although over 300 genetic variants are known to be associated with prostate cancer risk, few have been associated with the risk of an aggressive disease. One such variant is rs77559646 located in *ANO7*. This variant has a dual function. It constitutes a missense mutation in the short isoform of ANO7 and a splice region mutation in full-length *ANO7*. In this study, we have analyzed the impact of the variant allele of rs77559646 on *ANO7* mRNA splicing using a minigene splicing assay and by performing splicing analysis with the tools IRFinder (intron retention finder), rMATS (replicate multivariate analysis of transcript splicing) and LeafCutter on RNA sequencing data from prostate tissue of six rs77559646 variant allele carriers and 43 non-carriers. The results revealed a severe disruption of *ANO7* mRNA splicing in rs77559646 variant allele carriers. Immunohistochemical analysis of prostate samples from patients homozygous for the rs77559646 variant allele demonstrated a loss of apically localized ANO7 protein. Our study is the first to provide a mechanistic explanation for the impact of a prostate cancer risk SNP on ANO7 protein production. Furthermore, the rs77559646 variant is the first known germline loss-of-function mutation described for *ANO7*. We suggest that loss of ANO7 contributes to prostate cancer progression.

## Introduction

Prostate cancer is the most common cancer and the third most common cause of death among European men (1). Studies demonstrating the influence of family history have shown prostate cancer being among the most heritable of human cancers with 57% of the risk attributed to genetic factors (2). More than 300 genetic variants have been associated with prostate cancer risk (3,4). However, these SNPs (single nucleotide polymorphism) mostly show little or no ability to discriminate between indolent and fatal forms of the disease. In contrast to the high genetic component accounting for the overall prostate cancer risk, the heritability of prostate cancer-specific survival was estimated to be 10% (5) and consequently, few genetic variants have been specifically associated with aggressive prostate cancer (6,7). Since the majority of all diagnosed prostate cancer cases do not progress to lethality, biological markers that can distinguish between potentially lethal and non-aggressive cases would greatly improve prognostication and guide treatment decisions.

We have recently identified two SNPs, rs77559646 (NC_000002.12:g.241195850G > A, NP_001001666.2:p.Arg 104His) and rs148609049 (NC_000002.12:g.241188699C >  T), in *ANO7* [*TMEM16G*, *NGEP* (*New Gene Expressed in Prostate*), *D-TMPP* (*Dresden-transmembrane protein of the prostate*)] that are associated with an increased risk for aggressive prostate cancer (8). Furthermore, rs77559646 G > A was associated with a favorable response to docetaxel (9). ANO7 belongs to the anoctamin family of membrane proteins (ANO1–10), which function as Ca^2+^-activated Cl^−^ channels or lipid scramblases (10,11). Current knowledge on ANO7 function in cells is limited, and its function in the prostate remains to be elucidated. Like the majority of the family members, ANO7 has scramblase activity (12), but results regarding its ion channel function have been conflicting (13). Functional studies based on overexpression have implicated ANO7 in cell–cell adhesion (14) and vesicle trafficking (15). *ANO7* was initially characterized as a prostate-specific gene (16), with a very low level of transcript detectable in several other tissues (17). Two high confidence reference transcripts have been described. Full-length *ANO7* (NM_001370694.2; *ANO7-L*) consists of 25 exons, whereas the short isoform (NM_001001666.4; *ANO7-S*) arises via alternative cleavage and polyadenylation in intron 4. Consequently, the coding sequence of the last exon of ANO7-S extends into the intron (14,16). Several studies have suggested that both *ANO7* mRNA (18–21) and protein (22,23) are downregulated in advanced cancer, and that low *ANO7* expression predicts biochemical recurrence and metastatic progression (19–22). However, a study by Das *et al*. (24) found no correlation between grade and ANO7 protein expression, whereas in our own study, high *ANO7* mRNA expression correlated with a poor prognosis (8).

The *ANO7* variant rs77559646 G > A has a potential dual effect. Being localized in the unique portion of ANO7-S encoded by the 5′ part of intron 4, it has been referred to as a missense mutation (25). However, since this site resides within the splice region downstream of exon 4, it potentially also affects splicing of *ANO7-L* mRNA. The aim of this study was to analyze the impact of the variant allele of rs77559646 on *ANO7* mRNA splicing. Using both a minigene splicing assay and RNA sequencing (RNA-Seq) data from rs77559646 variant carriers analyzed using genome-wide splicing analysis tools, we could confirm that rs77559646 disrupts splicing. Furthermore, immunohistochemical analyses of prostate tissue sections from homozygous rs77559646 variant carriers revealed a striking loss of ANO7 protein. We propose that ANO7 acts as a tumor suppressor in the prostate.

## Results

### The A allele of rs77559646 causes exon skipping in a minigene splicing assay

**Figure 1:**
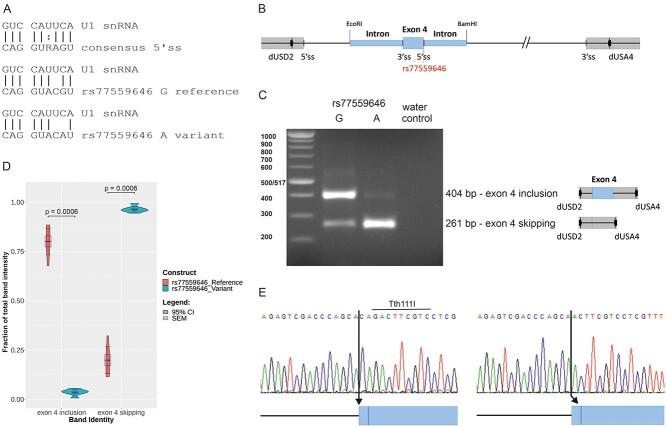
The A allele of rs77559646 causes exon skipping in a minigene splicing assay. (**A**) Base pairing between U1 snRNA and 5′ splice site sequences. (**B**) Schematic view of exon 4 with upstream and downstream intron sequences (blue boxes) cloned into pSPL3. The 5′ and 3′ splice sites, the position of rs77559646 and the primer binding sites for dUSD2 and dUSA4 are denoted. (**C**) A representative image of the minigene splicing assay RT-PCR amplification products following transfection of a construct carrying the G allele (left lane) or the A allele (right lane). The amplified products are schematically shown next to the gel image. (**D**) Quantification of the band intensities. The statistics is calculated from seven PCR-reactions derived from three independent transfections with similar results. Exon 4 inclusion reference: mean 0.801, standard error of the mean (SEM) 0.028, 95% CI 0.068. Exon 4 inclusion variant: mean 0.036, SEM 0.006, 95% CI 0.014. Exon 4 skipping reference: mean 0.199, SEM 0.028, 95% CI 0.068. Exon 4 skipping variant: mean 0.964, SEM 0.006, 95% CI 0.014. (**E**) Sanger sequencing of exon 4 inclusion RT-PCR products. The vector splice donor has been spliced to the upstream splice acceptor in intron 3 (left), or to the downstream splice acceptor three nucleotides downstream (right). The cleavage site for Tth111I is marked.

The *ANO7* variant rs77559646 G > A is located in the 5′ splice region five nucleotides downstream of exon 4. In the first step of the splicing process, the −3 to +6 region of the exon/intron junction is base paired with the 5′ terminus of U1 small nuclear RNA [snRNA; (26)]. The reference allele of rs77559646 deviates from the consensus 5′ splice site at position +4, and the variant allele A at position +5 introduces one additional mismatch with U1 snRNA (Fig. 1A). The G > A replacement is predicted to reduce the splicing efficiency of the splice site, as the Maximum Entropy (MaxEnt) score (i.e. log-likelihood ratio) decreases from 10.65 to 7.79 (Supplementary Material, Table S1), and the splice site strength by 18.7% according to Alamut. To verify this prediction, we used a minigene splicing assay. In this assay, a variant predicted to disrupt splicing is introduced into an artificial background of vector-provided exons, introns and splice donor and acceptor sites. ANO7 exon 4 with surrounding intronic sequences was cloned into the expression vector pSPL3 (Fig. 1B). Constructs carrying either the reference G or the variant A allele were transfected into cells, and the resulting RNA was analyzed by reverse transcription polymerase chain reaction (RT-PCR). The result showed that with the reference G allele, 80% of the PCR product corresponded to correctly spliced RNA, i.e. including exon 4, whereas 20% was the result of exon skipping, i.e. splicing between the vector donor and acceptor sites. In contrast, the variant A allele resulted in 96% exon skipping, and only 4% of the PCR product came from a properly spliced RNA (Fig. 1C and D). The identities of the PCR products were confirmed by cloning and sequencing of individual clones representing the major product produced by each construct, i.e. the larger product derived from the G allele and the smaller product derived from the A allele. Sequencing of the larger product revealed usage of two different splice acceptors located 3 bp (base pair) apart at the intron 3/exon 4 junction (Fig. 1E). Analysis of 25 clones by sequencing or restriction digestion using the enzyme Tth111I, which cleaves only clones spliced at the upstream site, revealed 12 clones spliced at the upstream site and 13 clones at the downstream site. In conclusion, these results confirm that the A allele of rs77559646 has a strong negative impact on splicing of *ANO7* exon 4.

### The A allele of rs77559646 increases intronic retention around exon 4 in the prostate

**Figure 2:**
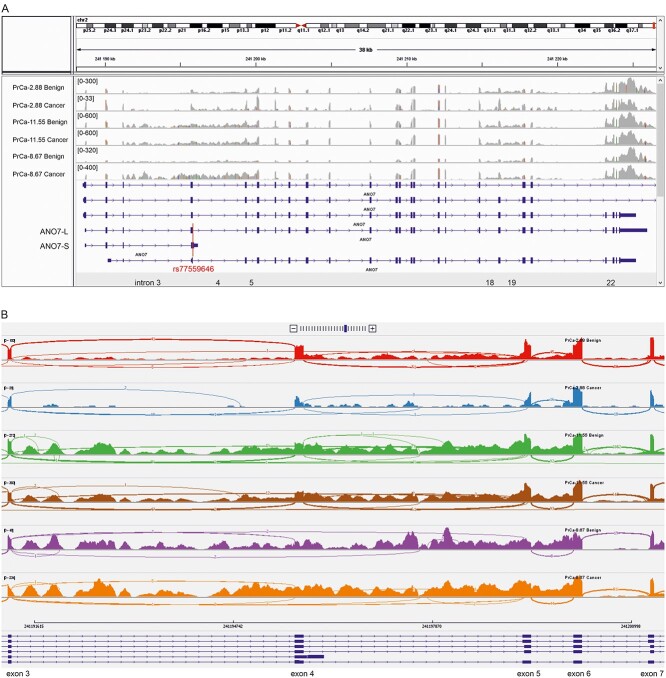
RNA-Seq from prostate tissue reveals aberrant RNA splicing in the exons 3–5 region of *ANO7*. Benign and cancer prostate tissue from a non-carrier (PrCa-2.88), a heterozygous carrier (PrCa-11.55) and a homozygous carrier (PrCa-8.67) are shown. The coverages shown as bar graphs are not to scales between samples. (**A**) RNA read coverage across the *ANO7* gene. The reference transcripts corresponding to *ANO7-L* and *ANO7-S* as well as the introns 3–5, 18, 19 and 22 are marked. The red line across the transcripts marks the position of rs77559646. (**B**) Sashimi plots across the exons 3–7 region. The arcs represent the splice junctions, the line thickness increase with the number of reads (indicated) split across the junction.

To investigate the impact of the A allele of rs77559646 on endogenous *ANO7* mRNA splicing, we utilized an RNA-Seq data set consisting of matched benign and tumor samples from 49 prostate cancer patients (27). Six germline carriers of the rs77559646 variant allele (hereafter denoted ‘carrier’), five heterozygotes and one homozygote, were included in the data set. Visual inspection of the RNA read alignments revealed that all carriers, but none of the non-carriers, exhibited a high level of *ANO7* intron 3 retention (Fig. 2, Supplementary Material, Fig. S1). Introns 4 and 5, as well as introns 18, 19 and 22, were also quite highly retained, but in contrast to the intron 3 retention, this was observed to a varying degree in all prostate samples. Interestingly, the MaxEnt score of the intron 4 3′ splice site is only 1.53, indicating a low splicing efficiency of this intron (Supplementary Material, Table S1).

To quantitate the difference in intron retention between carriers and non-carriers, we utilized the tool IRFinder [intron retention finder; (28)], which detects differential intron retention on a genome-wide level between sample groups. The results confirmed that the above-mentioned introns were retained at an average level of over 20% in all groups (Supplementary Material, Table S2). In the comparison between carriers and non-carriers, only one intron was found to be significantly differently retained on the genome-wide level in both the benign and cancer condition; intron 3 in *ANO7* (intron retention change, IRchange 0.295, *q* = 3.90 × 10^−7^and IRchange 0.323, *q* = 1.70 × 10^−4^, respectively). Introns 4 and 5 were also retained at a higher level in carriers compared to non-carriers in both the benign (IRchange 0.305 and 0.241, respectively) and cancer (IRchange 0.275 and 0.214, respectively) conditions, but this difference was not statistically significant. Introns 18, 19 and 22 were retained at a similar level in carriers and non-carriers (Table 1).

**Table 1:** IRFinder results on differentially retained *ANO7* introns between carriers and non-carriers

	**Benign**					**Cancer**				
**Intron**	**log2FoldChange**	**lfcSE**	***P***	***q***	**IRchange**	**log2FoldChange**	**lfcSE**	***P***	***q***	**IRchange**
3 (Upstream 3′ss)	3.584	0.52	5.60 × 10^−12^	3.90 × 10^−7^	0.295	3.786	0.64	3.40 × 10^−9^	1.70 × 10^−4^	0.323
3 (Downstream 3′ss)	3.584	0.52	5.60 × 10^−12^	3.90 × 10^−7^	0.295	3.786	0.64	3.30 × 10^−9^	1.70 × 10^−4^	0.323
4	1.956	0.567	5.60 × 10^−4^	1	0.305	1.748	0.728	0.016	1	0.274
5	1.561	0.537	3.70 × 10^−3^	1	0.241	1.381	0.706	0.05	1	0.215
18	0.143	0.52	0.784	1	0.018	0.048	0.661	0.942	1	0.006
19	0.128	0.52	0.805	1	0.021	0.09	0.663	0.892	1	0.015
22	−0.026	0.504	0.958	1	−0.004	0.013	0.675	0.984	1	0.002

Gray fields show non-significant results.

3’ss = 3′ splice site.

### Several aberrant splicing events are detected in rs77559646 A allele carriers

Disruption of splicing often results in exon skipping and cryptic splicing (29), and for analysis of these events in ANO7, we utilized the tools rMATS [replicate multivariate analysis of transcript splicing; (30)] and LeafCutter (31), which detect significant splicing changes between groups on a genome-wide level. These tools differ in that rMATS analyzes every splicing event separately, whereas LeafCutter clusters overlapping introns that share a donor or acceptor splice site and then conducts a differential analysis on these clusters.

Following removal of low-evidence splicing events, rMATS detected 400 unique events in *ANO7* with a minimum splicing change of 5% between the groups (Supplementary Material, Table S3). For the cancer condition, only one of these events was statistically significant; skipping of exon 4. On the group level, the inclusion of exon 4 was reduced in the carriers compared to the non-carriers [inclusion level difference − 0.153, false discovery rate (FDR) 0.026]. For the benign condition, four statistically significant events were detected. Three of the events were exon skipping events, describing cryptic exons in intron 3. These cryptic exons were included to a higher extent in carriers than in non-carriers (inclusion level differences 0.173, 0.249 and 0.199, respectively; FDR 0). The fourth statistically significant event was the increased usage of an alternative 5′ splice site in intron 7 in the carrier group (Table 2). The same event was also detected by LeafCutter (Table 3).

**Table 2:** rMATS results on differentially spliced events in *ANO7* between carriers and non-carriers

**Event description**	**Benign**				**Cancer**			
	**Event ID**	**IncLevelDifference**	***P*-value**	**FDR**	**Event ID**	**IncLevelDifference**	***P*-value**	**FDR**
Skipped exon: exon 4 reference. Upstream exon: exon 3 extended in the 5′ end with 9 nucleotides. Downstream exon: exon 5 reference.	12 722	−0.166	2.82 × 10^−2^	1	12 384	−0.153	1.75 × 10^−6^	2.56 × 10^−2^
Skipped exon: Cryptic exon in intron 3 (153 bp, 241 191 763–241 191 915). Upstream exon: exon 3 reference. Downstream exon: exon 4 ANO7-S reference.	12 612	0.173	0.00	0.00	Not detected			
Skipped exon: cryptic exon in intron 3 (189 bp, 241 194 842–241 195 030). Upstream exon: exon 3 reference. Downstream exon: exon 4 truncated in the 5′ end with 3 nucleotides.	12 627	0.249	0.00	0.00	Not detected			
Skipped exon: cryptic exon in intron 3 (247 bp, 241 191 669–241 191 915). Upstream exon: exon 3 reference. Downstream exon: exon 4 truncated in the 5′ end with 3 nucleotides.	12 688	0.199	0.00	0.00	Not detected			
Long exon: exon 7 extended in the 3′ end with 169 nucleotides. Short exon: exon 7 reference. Flanking exon: exon 8 reference.	3021^*^	0.162	1.30 × 10^−9^	2.59 × 10^−5^	3044^*^	0.139	1	1
Long exon: exon 7 extended in the 3′ end with 169 nucleotides. Short exon: exon 7 reference. Flanking exon: exon 8 reference.	3021^*^	0.158	8.20 × 10^−9^	3.25 × 10^−4^	3044^*^	0.141	1	1

^^*^^Event 3021/3044 results from both ‘ReadsOnTargetAndJunctionCounts’ and ‘JunctionCountsOnly’ are reported.

Gray fields show non-significant results.

**Table 3:** LeafCutter results on differentially spliced clusters in *ANO7* between carriers and non-carriers

**Exons 3–5**	**Cancer**					**Benign**							
**Cluster data**	**Number of junctions**	**Loglr**	***P***	***q***		**Number of junctions**	**Loglr**	***P***	***q***				
	12	60.605	1.04 × 10^−20^	9.62 × 10^−17^		13	21.18	2.90 × 10^−5^	8.60 × 10^−2^				
**Junction data**	**logef**	**Fraction of junctions in non-carrier**	**Fraction of junctions in carrier**	**ΔPSI**	**Rank**	**logef**	**Fraction of junctions in non-carrier**	**Fraction of junctions in carrier**	**ΔPSI**	**Rank**	**Start**	**End**	**Junction description** ^*^
	3.018	0.002	0.071	6.93 × 10^−2^	a	−0.232	0.002	0.002	−6.85 × 10^−5^	h	241 191 251	241 199 316	Exon 3 > exon 5
	−0.822	0.472	0.422	−5.00 × 10^−2^	b	−0.232	0.471	0.458	−1.31 × 10^−2^	b	241 191 251	241 195 706	Exon 3 > internal exon 4, 3 nt missing from the 5′ end of the exon^*^
	−0.822	0.432	0.386	−4.59 × 10^−2^	c	−0.232	0.429	0.417	−1.19 × 10^−2^	c	241 195 845	241 199 316	Exon 4 > exon 5^*^
	2.621	0.001	0.019	1.80 × 10^−2^	d	−0.232	0.002	0.002	−4.71 × 10^−5^	h	241 191 251	241 197 672	Exon 3 > intron 4, 1827 nt downstream of exon 4
	1.593	0.002	0.016	1.40 × 10^−2^	e	2.781	0.001	0.029	2.77 × 10^−2^	a	241 191 251	241 192 002	Exon 3 > intron 3, 751 nt downstream of exon 3
	−0.821	0.025	0.022	−2.65 × 10^−3^	f	−0.232	0.025	0.024	−6.92 × 10^−4^	d	241 195 845	241 199 319	Exon 4 > internal exon 5, 3 nt missing from the 5′ end of the exon
	−0.819	0.016	0.014	−1.63 × 10^−3^	g	−0.232	0.018	0.018	−5.10 × 10^−4^	d	241 195 845	241 199 363	Exon 4 > internal exon 5, 47 nt missing from the 5′ end of the exon
	−0.669	0.029	0.03	1.21 × 10^−3^	h	−0.231	0.03	0.029	−8.08 × 10^−4^	d	241 191 251	241 195 703	Exon 3 > exon 4^*^
	−0.821	0.014	0.012	−1.44 × 10^−3^	i	−0.232	0.01	0.01	−2.81 × 10^−4^	d	241 195 845	241 197 672	Exon 4 > intron 4, 1827 nt downstream of exon 4
	−0.816	0.003	0.003	−3.34 × 10^−4^	j	−0.232	0.003	0.003	−8.94 × 10^−5^	h	241 195 845	241 199 288	Exon 4 > intron 4, 28 nt upstream of exon 5
	−0.821	0.004	0.004	−4.61 × 10^−4^	j	−0.232	0.005	0.005	−1.40 × 10^−4^	h	241 197 793	241 199 316	Intron 4, 1948 nt downstream of exon 4 > exon 5
	−0.82	0.001	0.001	−9.06 × 10^−5^	j	−0.232	0.001	0.001	−3.54 × 10^−5^	h	241 197 793	241 199 319	Intron 4, 1948 nt downstream of exon 4 > internal exon 5, 3 nt missing from the 5′ end of the exon
	NA	NA	NA	NA	NA	−0.231	0.001	0.001	−2.95 × 10^−5^	h	241 191 251	241 194 842	Exon 3 > intron 3, 3591 nt downstream of exon 3
**Exon 7**	**Cancer**					**Benign**							
**Cluster data**	**Number of junctions**	**Loglr**	***P***	***q***		**Number of junctions**	**Loglr**	***P***	***q***				
	3	7.935	3.58 × 10^−4^	1.89 × 10^−1^		2	9.877	8.81 × 10^−6^	3.64 × 10^−2^				
**Junction data**	**logef**	**Fraction of junctions in non-carrier**	**Fraction of junctions in carrier**	**ΔPSI**	**Rank**	**logef**	**Fraction of junctions in non-carrier**	**Fraction of junctions in carrier**	**ΔPSI**	**Rank**	**Start**	**End**	**Junction description**
	−0.387	0.905	0.753	−1.51 × 10^−1^	b	−0.63	0.902	0.723	−1.79 × 10^−1^	a	241 201 355	241 202 194	Exon 7 > exon 8^*^
	0.774	0.091	0.243	1.52 × 10^−1^	a	0.63	0.098	0.277	1.79 × 10^−1^	b	241 201 524	241 202 194	Intron 7, 169 nt downstream of exon 7 > exon 8
	−0.387	0.004	0.003	−6.63 × 10^−4^	c	NA	NA	NA	NA	NA	241 201 810	241 202 194	Intron 7, 455 nt downstream of exon 7 > exon 8

^^*^^Annotated reference junctions.

Gray fields show non-significant results.

**Figure 3:**
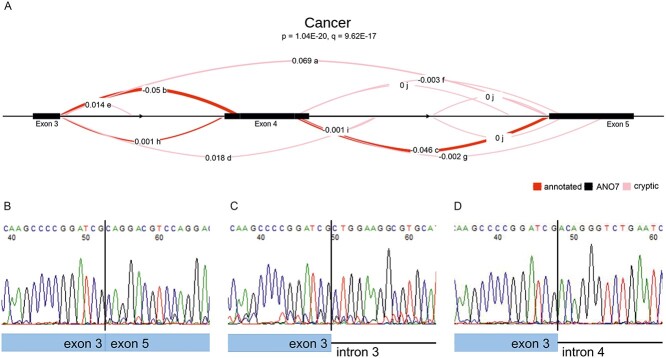
Splicing cluster in *ANO7* identified by LeafCutter to be significantly differentially spliced in carriers compared to non-carriers. (**A**) The exons 3–5 cluster differs among the cancer samples. The original ‘Fraction of junction’ values printed by the LeafCutter’s LeafViz R shiny application have been manually replaced with ΔPSI (percent spliced in) values. The letters a-j denote the ranking of the events performed by the tool. (**B**–**D**) Sanger sequencing of RT-PCR products across aberrant splicing events. (B) Splicing from exon 3 to exon 5, junction a. (C) Splicing from exon 3 to position 241 192 002 in intron 3, junction e. (D) Splicing from exon 3 to position 241 197 672 in intron 4, junction d.

Also LeafCutter detected significant splicing changes around exon 4 in ANO7. A splicing cluster between exons 3 and 5 consisting of 12 separate splicing events was determined to be significantly (*q* = 9.62 × 10^−17^) differentially spliced between carriers and non-carriers in the cancer condition. In the benign condition, the difference was not significant (*q* = 8.60 × 10^−2^; Fig. 3A, Table 3). For the carrier group, 9% of the spliced reads in the region skipped exon 4 entirely, either by splicing directly from exon 3 to exon 5 or from exon 3 to a cryptic splice site in intron 4 (position 241 197 672). In the non-carriers, only 0.3% of the spliced reads skipped exon 4. The carriers also had a proportionally higher number of reads splicing from exon 3 into intron 3 (position 241 192 002), the proportion of all spliced reads being 1.6% in carriers compared to 0.02% in non-carriers. As a result of these aberrant splicing events, there was a comparative decrease in annotated normal splicing for carriers, 83.8%, compared with 93.3% in the non-carriers. Interestingly, the major site utilized for intron 3 removal was the downstream acceptor site, accounting for over 40% of the splice junctions in all groups. The upstream acceptor site was used in only 3% of the junctions. The few reads spliced from exon 3 to exon 4 observed in the homoygous cancer sample were also mostly to the downstream site (one and five reads, respectively; Fig. 2B).

For validation using RT-PCR and sequencing, we designed primers for detection of the three most differentially spliced aberrant splice junctions as reported by LeafCutter. Total RNA isolated from 22Rv1 cells or from normal prostate tissue, both rs77559646 carriers, was used as template. All three splice junctions were detected in prostate RNA, i.e. exon 3 spliced to exon 5, exon 3 spliced to position 241 197 672 in intron 4 and exon 3 spliced to 241 192 002 in intron 3 (Fig. 3B–D). In 22Rv1 cells, exon 4 skipping was detected (results not shown).

### The rs77559646 carriers display allelic imbalance of intron 3, 4 and 5 retention, but not of expression of upstream and downstream exonic sequences

Most of the aberrant splicing events detected are predicted to result in mRNAs containing premature stop codons (Supplementary Material, Supplementary information), which activates the nonsense-mediated decay (NMD) pathway and degradation of the mRNA (32). If the two alleles in the heterozygous carriers are transcribed at an equal level, we expect that NMD will reduce the fraction of the mRNA coming from the allele carrying the variant A allele at rs77559646. Consequently, we will observe an allelic imbalance in the RNA-Seq data across the *ANO7* gene. Furthermore, IRFinder indicated an increased retention of introns 4 and 5 in carriers compared to non-carriers. An analysis of the allele frequencies of any intronic SNPs located in introns 4 and 5 in RNA-Seq will reveal whether or not these introns are affected by the splicing defect observed in the rs77559646 carriers.

To analyze the allelic imbalance, we first extracted altogether 204 germline SNPs in the ANO7 region from blood WGS (whole genome sequencing) data obtained from 39 prostate cancer patients (including seven heterozygous and two homozygous carriers) and three bladder cancer patients (including one heterozygous carrier; Supplementary Material, Table S4). The homozygous rs77559646 carrier PrCa-8.67 proved to be homozygous for essentially the whole ANO7 region, while the second homozygote, PrCa-10.48, was homozygous only for the exons 1–15 region. The alleles of the haplotype carried by PrCa-8.67 were, with a few exceptions, shared by all rs77559646 carriers in either homozygous or heterozygous condition. The frequencies of the alleles carried by PrCa-8.67 were therefore quantified in the RNA-Seq for the nineteen patients with known genotypes and used in all subsequent calculations.

**Figure 4:**
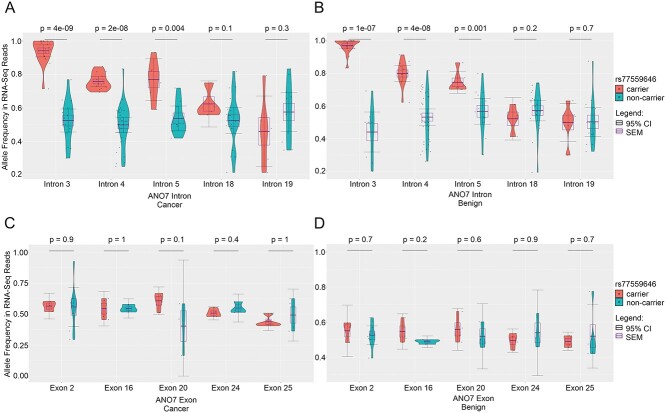
*ANO7* allele frequencies in RNA-Seq from carrier and non-carrier prostate tissue. (**A**, **B**) The rs77559646-linked alleles located in introns 3–5, but not in introns 18 and 19, are expressed at significantly higher frequencies in carriers than in non-carriers in cancer (A) and benign (B) conditions. (**C**, **D**) The rs77559646-linked alleles in selected exons are expressed at equal frequencies in carriers and non-carriers in cancer (C) and benign (D) conditions. *N*, standard deviation (sd), mean, SEM and 95% CI values for each group of SNPs are listed in Supplementary Material, Table S5.

Between group comparisons of pooled SNPs in introns 3, 4 and 5 showed that the presumed rs77559646-linked alleles were expressed at a significantly higher frequency in carriers (range 0.75–0.97) than in non-carriers (range 0.44–0.57), whereas there was no difference between the groups of the mean allele frequencies of SNPs located in introns 18 and 19 (range 0.46–0.62) (Fig. 4A and B, Supplementary Material, Table S5).

Four individual exonic SNPs located in exons 16, 20, 24 and 25 had sufficient data to be analyzed individually, whereas SNPs in exon 2 were pooled. The average group-wise frequencies of the presumed rs77559646-linked SNPs ranged from 0.40 to 0.61 and were not significantly different between carriers and non-carriers (Fig. 4C and D, Supplementary Material, Table S5).

These results support the conclusion that the A allele of rs77559646 causes increased retention of introns 4 and 5 in addition to intron 3. However, this deviation from the normal splicing pattern does not affect the balance between the alleles in the 5′ or 3′ part of the gene, indicating that *ANO7* mRNA derived from the variant allele is not degraded to a significant extent.

### The A allele of rs77559646 results in loss of apical ANO7 protein expression

We identified altogether four rs77559646 homozygotes in our collection of 946 genotyped prostate cancer patients (Table 4, Supplementary Material, Table S6). Any ANO7 protein produced in these patients is encoded by a gene carrying the splice site mutation, which, as shown previously, results in the expression of grossly abnormal mRNA. If these mRNAs escape degradation and enter the translational machinery, they will be translated into truncated protein products that lack all transmembrane domains (Supplementary Material, Supplementary information), and will therefore be unable to insert into the plasma membrane. Previous studies have shown that full-length ANO7 protein localizes apically and laterally in the benign prostatic epithelium (14,23,24,33,34), and that the expression is reduced in high grade prostate cancer compared to low grade cancer and normal prostate epithelium (22,23). To analyze the effect of the rs77559646 variant on ANO7 protein expression, we selected areas containing benign prostate tissue derived from the four homozygous carriers and performed immunohistochemistry with a polyclonal antibody that recognizes the N-terminal part of ANO7. As a control, a benign sample from a non-carrier (PrCa-2.88) was selected, that according to the RNA-Seq data expressed an equal level of *ANO7* as the homozygous carrier PrCa-8.67.

**Table 4:** Clinical characteristics of homozygous rs77559646 carriers

**Patient ID**	**Age at diagnosis**	**Preoperative PSA**	**Gleason grade**	**pT-class**	**pN-class**	**M-class**
PrCa-8.67	67	5.5	4 + 5	T3b	N1	M0
PrCa-10.48	66	11	4 + 5	T3b	N0	M0
PrCa-17.11	66	3.1	3 + 4	T2	NX	M0
PrCa-34.84	73	12	3 + 4	T2c	NX	M0

PSA, prostate-specific antigen, ng/ml;

pT, pathologic tumor;

pN, pathologic lymph node;

M, metastasis;

PrCa, prostate cancer.

**Figure 5:**
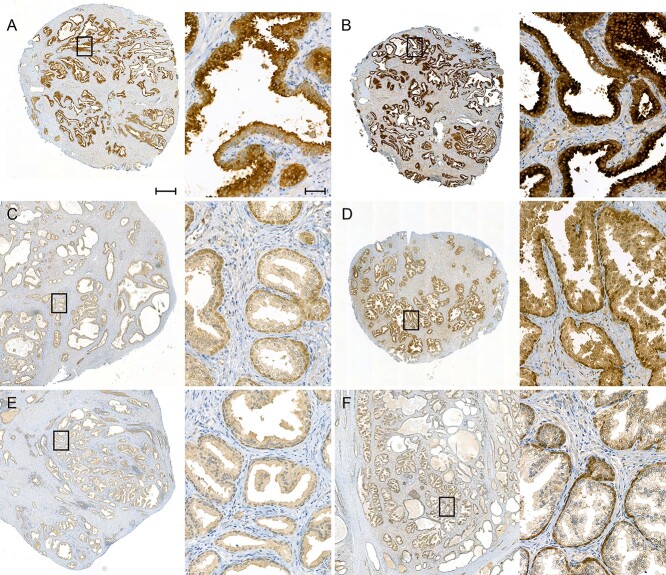
Apical ANO7 protein expression is lost in homozygous rs77559646 carriers. Benign prostate tissue stained with an N-terminal anti-ANO7 antibody. The left image is captured at 2× (scale bar 50 μm) and the right image at 20× (scale bar 500 μm) magnification. (**A**, **B**) In the non-carrier (PrCa-2.88), ANO7 is strongly accumulated at the apical plasma membrane of the luminal cells in the prostatic epithelium. The two samples were stained at different time points with minor reagent changes. (**C**–**F**) Benign tissue from homozygous rs77559646 carriers. No ANO7 accumulation is observed at the apical plasma membrane. The epithelial cells are more weakly and evenly stained, and perinuclear staining in the basal cells appear. (C) PrCa-8.67, the ANO7 mRNA level in this sample is similar to the one in the non-carrier in (A, B). (D) PrCa-17.11. (E) PrCa-10.48. (F) PrCa-34.84, this sample was processed and fixed according to a different protocol. (A) and (C) were stained in the same batch, (E) in a different batch but with the same reagents. (B, D) and (F) were stained in the same batch.

As expected, the non-carrier sample exhibited a prominent staining at the apical plasma membrane in the benign prostatic ducts (Fig. 5A and B). In contrast, the prostatic ducts in the homozygous carriers were essentially devoid of apical ANO7 staining. Instead, a weaker and even staining throughout the prostatic epithelial cells was seen, along with a stronger perinuclear staining in the basal cells (Fig. 5C–F). The diffuse signal in the carriers may be derived from truncated protein products recognized by the antibody, and the appearance of expression in the basal cells may imply a more complicated change in the *ANO7* gene regulation due to the mutation. An alternative explanation is that the antibody signal arises from cross-reacting epitopes (https://v18.proteinatlas.org/ENSG00000146205-ANO7/antibody and Supplementary Material, Fig. S2) emphasized in the ANO7 mutant background. In conclusion, the G > A mutation at rs77559646 acts via its effect on splicing and results in a loss of full-length ANO7 protein.

## Discussion

*ANO7* has been associated with prostate cancer in a large number of studies, and several risk SNPs in *ANO7* have been identified (25). The A allele of rs77559646 is associated with both risk and aggressiveness of prostate cancer (8). The G > A mutation of rs77559646 is a dual function mutation, in that it acts as a splice site mutation in relation to *ANO7-L* and as a missense mutation in ANO7-S. To the best of our knowledge, no studies on the endogenous ANO7-S have yet been presented, and there is no published data about the relative levels of *ANO7-L* and *ANO7-S* mRNA in prostate tissue. In this work, we have shown that the A allele of rs77559646 has a dramatic impact on *ANO7-L* mRNA splicing. *ANO7-S* is the result of polyadenylation and cleavage in intron 4, and thus would not depend on correct removal of intron 4. However, removal of intron 3 is also severely affected by the variant allele. Furthermore, four patients confirmed to be homozygous carriers of this allele all showed a severe reduction of ANO7 protein at the apical plasma membrane of the prostatic luminal epithelium. The antibody detects both ANO7-L and ANO7-S, and if the mis-spliced *ANO7-L* mRNAs simply would have been converted into *ANO7-S* mRNA and protein production, the staining intensity had remained high despite the loss of apical staining. Therefore, we do not believe that the expression of a mutated ANO7-S protein is the main cause of the increased risk of aggressive prostate cancer. Instead, we propose that the risk is due to a severe loss of ANO7-L because of aberrant splicing. Still, an additional negative impact caused by a low level of a mutated ANO7-S protein cannot be ruled out.

A substantial proportion of human disease-causing mutations have been found to act by disrupting mRNA splicing (29). The rs77559646 variant is a G > A transition at the +5 position in the 5′ splice site in intron 4. This position is the second most commonly affected splice-altering mutation among disease-causing germline mutations (35) and it is also common among somatic mutations in cancer (36). *In silico* analysis of the rs77559646 variant predicted an 18.7% reduction in splice site strength, while the minigene splicing assay demonstrated nearly complete exon skipping. The results from the prostate RNA-Seq data analyses revealed a more complicated picture. The most striking difference was a large increase in *ANO7* intron retention in rs77559646 carriers, also detected by the IRFinder. This result was somewhat unexpected, since mutations in the splice donor site most commonly results in exon skipping or activation of cryptic splicing (29,35). Full intron retention is much less common, although it has been detected in patient RNA (37,38). However, since our analysis is based on RNA-Seq data consisting of short reads, the level of full intron retention is only computationally estimated. The differential alternative splicing analyses conducted using rMATS and LeafCutter further demonstrated that the splicing pattern of the exons 3–5 region in rs77559646 carriers differed significantly at the genome-wide level from the one seen in non-carriers, involving both exon 4 skipping and activation of cryptic splice sites. As five of the six carriers were heterozygotes, the analyzed RNA is a combination of normally and aberrantly spliced ANO7. Therefore, the true magnitude of the splicing defect due to the mutation is larger than estimated by these tools.

The exact molecular mechanism behind the failed splicing induced by the A allele of rs77559646 remains to be identified, although reduced binding between the splice donor site and the U1 snRNA is most likely involved. The observed consequences on splicing in the exon 4 region can be explained via the exon definition theory (39), according to which the spliceosomal small nuclear ribonucleoproteins assembled at either side of the exon communicate with one another. A mutation in the 5′ splice site may affect the assembly of the splicing machinery also at the upstream 3′ splice site, particularly in cases with weak 3′ splice sites, causing failed splicing of the upstream intron as well (40). Similarly, a weak 3′ splice site downstream of the mutated 5′ splice site can lead to failed splicing of the downstream exon (41). The very low splice site strength of the 3′ splice site of intron 4 suggests that exon 5 is weakly defined, which may lead to less efficient intron 4 and 5 removal, as observed in all sequenced prostate cancer samples regardless of genotype. This feature of exon 5, in combination with loss of the intron 4 5′ splice site due to the rs77559646 variant, might explain the enhanced retention of intron 5 in carriers.

In most cases, intron retention introduces premature stop codons in the mRNA. Translation of such mRNAs results in the production of potentially deleterious truncated protein products. Normally, this is prevented by the activation of NMD, which rapidly reduces the level of intron-containing mRNA (32). Apart from being a consequence of defective splicing, intron retention and degradation also functions as a mechanism for a regulated reduction of transcript and protein levels in the cell (42). However, mature transcripts that contain introns may also be stored intact in the nucleus for later regulated splicing in response to various signals (43–45). A large number of genes, including *ANO7*, have previously been shown to contain one or several introns that are significantly retained (42). The observation presented in this work that certain *ANO7* introns are more retained is in line with these findings. The A allele of rs77559646 results in an even higher level of intron retention, yet the allele specific expression analysis showed that equal levels of mRNA were expressed from the reference and the variant allele. This indicates that mutant *ANO7* mRNA does not undergo degradation to a significant extent, and raises the question of whether a major fraction of the *ANO7* mRNA is actually retained in the nucleus. If only a minor fraction of the *ANO7* mRNA resides in the cytoplasm, NMD-mediated degradation of transcripts derived from the variant allele of rs77559646 might not have a detectable impact on the allelic ratios when total mRNA is analyzed. The results presented here warrants a closer examination of *ANO7* mRNA localization in prostate cells.

One out of the four identified rs77559646 homozygous carriers was included in the RNA-Seq cohort. The prostate tissue samples from this patient expressed readily detectable ANO7 mRNA across the whole gene, but nevertheless, ANO7 protein expression was dramatically reduced. This underscores the fact that the level of mRNA expression does not necessarily correlate with the level of protein expression (28). Surprisingly, no morphological abnormalities had been detected in routine histopathological examination of non-cancerous tissue in the homozygotes at the time of diagnosis. It is not known if and how ANO7 is expressed during the early stages of prostate development. If ANO7 plays no role in prostate organogenesis, a morphologically normal prostate is expected to develop also in homozygous carriers of rs77559646. It is also possible that ANO7 function is redundant in the prostate, or that ANO7 mRNA derived from the variant allele is correctly spliced to a greater extent earlier in life, allowing a morphologically normal prostate to form. Marx *et al*. (22) demonstrated a strong independent prognostic role for ANO7 expression, and proposed that ANO7 indicates loss of polarity and dedifferentiation, rather than acting as a tumor suppressor. The fact that none of the homozygous rs77559646 carriers had been diagnosed at a particularly young age supports their view that loss of ANO7 does not drive tumor initiation. However, since a hereditary partial loss of ANO7 brings along an increased risk of aggressive prostate cancer, ANO7 is expected to exert some kind of anti-cancer function in the prostate. If such an anti-cancer function comes into play only after the cancer has initiated due to mutations elsewhere in the genome, it could explain why the homozygous rs77559646 carriers did not develop prostate cancer at a young age.

Analysis of ANO7 function has been severely hampered by the very low expression level (https://sites.broadinstitute.org/ccle/datasets) and apparent lack of ANO7 protein expression (our unpublished observations) in common prostate cancer cell lines. The western blot analyses on LNCaP cells (14) and on 22Rv1 cells (33) both demonstrated lack of ANO7 protein expression, despite detectable mRNA expression [Bera *et al*. (16); this study]. These two cell lines both represent advanced prostate cancer (46,47), stages in which ANO7 tends to be downregulated (18,22,23). Tumor progression data thus clearly includes downregulation of ANO7 expression, but does not reveal whether loss of ANO7 is a cause or a consequence of tumor progression. Because the risk SNP rs77559646 is now shown to cause loss of ANO7 protein, we propose that loss of ANO7 may actually contribute to tumor progression, i.e. that ANO7 acts as a tumor suppressor. The molecular mechanisms behind this remain to be discovered. A comprehensive transcriptome analysis of a larger cohort of homozygous rs77559646 carriers would likely provide valuable information regarding the role of ANO7 in the prostate.

In conclusion, we have presented evidence that the risk SNP rs77559646 results in loss of ANO7 protein. Accumulated evidence suggests that rs77559646 is a valuable marker for predicting prostate cancer risk and aggressiveness, and that it should be taken into account for guiding treatment decisions in the clinic.

## Materials and Methods

### Patient material

All prostate cancer samples analyzed were sourced from the Turku Prostate Cancer Consortium collection. The patients were diagnosed with localized prostate adenocarcinoma and underwent robot-assisted laparoscopic radical prostatectomy at Turku University Hospital. Forty-nine patients, including one homozygous and five heterozygous rs77559646 carriers, were subjected to RNA-Seq. Thirty-nine patients, including two homozygotes and seven heterozygotes, underwent WGS. One homozygote, 5 heterozygotes and 13 non-carriers were common for both cohorts. Three bladder cancer patients, one of these an rs77559646 carrier, were included in the WGS cohort. SNP genotyping identified two additional homozygous rs77559646 carriers, which were subjected to immunohistochemical analysis only. For RT-PCR analyses, prostate tissue from an rs77559646 carrier bladder cancer patient having undergone radical cystectomy was used. All the patients gave a written and signed informed consent according to the principles in the Declaration of Helsinki. The research was approved by the Institutional Review Board of the Turku University Hospital.

### Genotyping

Blood DNA was extracted using the kit BACC3 (GE Healthcare Life Sciences, Little Chalfont, UK) and genotyped for rs77559646 using TaqMan™ SNP Genotyping Assay and TaqMan™ Genotyping Master Mix (ThermoFisher Scientific, Waltham, MA, USA).

### Whole genome sequencing and variant calling

Blood DNA underwent whole genome sequencing at BGI Genomics in three batches. For the first two, a 350 bp insert library was sequenced as paired-end 150 bp reads on a Hiseq X Ten platform (Illumina). For the third, a BGISEQ Normal DNA library, was sequenced as paired-end 100 bp reads on a BGISEQ platform.

The raw DNA-Seq reads were inspected for quality with FastQC (https://www.bioinformatics.babraham.ac.uk/projects/fastqc/) and adaptor sequences were removed with cutadapt (48). Following the Genome Analysis Toolkit (GATK) best practices for version 4.0.11.0 (49,50), the pre-processed DNA-Seq reads were aligned to human Genome Reference Consortium Human build 38 genome assembly using BWA-MEM (Burrows-Wheeler Aligner Maximal Exact Match), version 0.7.17 (51), and coordinate sorted with Samtools, version 1.9(52). Sequencing duplicates were marked with Picard Tools, version 2.18.16 (Broad Institute). Base quality score recalibration (BQSR) was then performed with GATK.

Variant calling was performed using GATK, following the GATK best practises. HaplotypeCaller was used for initial variant calling and the genotypes were determined using the GenotypeGVCSs tool. Variant quality score recalibration (VQSR) was performed separately for single nucleotide variants (SNVs) and insertions/deletions (Indels) using the VariantRecalibrator and ApplyVQSR tools. In the VQSR step, the tranche thresholds 99.5 and 99.0 were used for SNVs and Indels, respectively, to determine true positive variants.

### RNA-Seq pre-processing and alignment

Tissue processing for RNA isolation and bulk sequencing of tumor and benign prostate tissue has been described (27). In brief, fresh frozen tumor samples next to formalin-fixed paraffin-embedded (FFPE) validation samples containing at least 50% of carcinoma were used. For morphologically benign prostatic tissue samples, the absence of carcinoma was similarly confirmed using parallel FFPE samples. The percentage of normal epithelium varied from 5 to 70% in the benign samples. Reads were subsequently pre-processed in accordance with rMATS v3.2.5 (30) requirements to remove poor quality 3′ ends and reads of variable length. Reads were then aligned with STAR (spliced transcripts alignment to a reference), version 2.5.2b (53), following rMATS’ recommended specifications for alignment, to National Center for Biotechnology Information’s (NCBI) GCA_000001405.15 ‘no alt plus hs38d1 analysis set’ genome alongside the Ensembl v95 annotation of the human genome.

### Differential splicing analyses

Differential splicing analyses were conducted for six rs77559646 carriers versus 43 non-carriers, separately for the cancer and benign conditions.

IRFinder v1.3.0 (28) was run with default parameters, using the generalized linear model mode. Significant (FDR < 0.05) differential intron retention events were identified, and the change in intron retention for each intron in the *ANO7* gene was examined. Multiple testing corrections were carried out using the Benjamini–Hochberg procedure.

We ran rMATS v3.2.5 with the novel splicing detection mode enabled, requiring a minimum splicing change of 5% between the sample groups. The output files for each splicing event type were filtered to remove low-evidence events, defined as having <10 reads in every sample for both event inclusion and skipping. FDR < 0.05 was considered statistically significant.

LeafCutter v0.2.9 (31) was run using default parameters. Significantly (FDR < 0.05) differentially spliced clusters were identified and plotted with LeafCutter’s LeafViz R shiny application.

### Allele frequency calculations

All germline variants in the *ANO7* region, here defined as 2:241188509–241 225 976, i.e. ranging from the start of NM_001001891.3 to the end of NM_001370694.2, were extracted from blood WGS data. The allele counts in the RNA-Seq of 204 SNPs were then quantified from the STAR alignments for the nineteen patients with known genotypes using the ‘bam-readcount’ tool (https://github.com/genome/bam-readcount). This produced a summary of high quality (base and alignment quality ≥ PHRED 20) allele frequencies at specified positions. To be included in the statistical analyses, at least 10 high quality reads were required at the position in question. The frequencies of the alleles present on the presumed rs77559646-linked haplotype were analyzed in R v3.6.0. Custom R scripts were written to analyze the allele frequencies and plot the results. Functions from the coin v1.3–0 (54) library were used for the statistical testing, and functions from the following libraries were used to organize the data and plot the results: ggplot2 v3.1.0 (55) (https://ggplot2.tidyverse.org), ggsignif v0.5.0 (https://github.com/const-ae/ggsignif), gridExtra v2.3 (https://github.com/cran/gridExtra), reshape2 v1.4.3 (56), Rmisc v1.5 (https://github.com/RyanHope/Rmisc). Individual exonic SNPs were analyzed if present in at least three samples in both the carrier and the non-carrier group. Exon 2 and introns 3–5, 18 and 19 were analyzed by pooling all SNPs for each exon or intron, respectively, and the average frequencies, error margins and 95% confidence intervals (CI) were calculated for each group of samples, i.e. cancer and benign in carriers and non-carriers. As these data were determined to be not normally distributed via the Shapiro–Wilk test, statistical difference (*P* < 0.05, two-tailed) in allele frequency distribution was determined using the Wilcoxon rank-sum test and population comparisons were calculated using Fisher’s exact test.

### Other *in silico* analyses

Integrative genomics viewer IGV_2.8.13 was used to visualize RNA-Seq read sequence coverage. The splice site analysis tool Alamut Visual v.2.7.1 was used with default settings. The MaxEnt scores for splice sites (57) were calculated using the webserver http://hollywood.mit.edu/burgelab/maxent/Xmaxentscan_scoreseq.html.

### Plasmid constructs

To create the constructs for the minigene splicing assay, DNA from the cell line 22Rv1, which is heterozygous for rs77559646, was used as template. Exon 4 and surrounding intronic sequences were amplified using the primers 5′-GGGGAATTCTTCTCTATGGCAGGACGGGA-3′ and 5′-GGGGGATCCGGAGACGGGACAGGTGAATG-3′. The primers included sites for EcoRI and BamHI. The resulting PCR-fragment was cloned into the plasmid pSPL3 (58), kindly provided by Dr Thomas van Overeem Hansen (Rigshospitalet, Copenhagen, Denmark). The insert of selected clones was confirmed with Sanger sequencing. All sequenced clones carried the following variant alleles in the upstream intron: rs10048694 (NC_000002.12:g.241195387G > A), rs12998792 (NC_000002.12:g.241195442A > G) and rs12694996 (NC_000002.12:g.241195602A > C). The clones carrying the variant allele at rs77559646 also carried the variant allele at rs147325507 (NC_000002.12:g.241196084C  > A) in the downstream intron. To create a construct only carrying the variant allele of rs77559646, a SacI-BamHI fragment in the variant construct was replaced with the corresponding fragment from the reference construct.

### Cell culture

An in-house stock of mycoplasma-negative COS-7 cells was grown in Dulbecco’s Modified Eagle Medium B12-709F (Lonza Group, Basel, Switzerland) supplemented with 2 mm UltraGlutamineTM I Supplement (Lonza Group), 10% volume per volume (v/v) fetal bovine serum (FBS) S1810 (Biowest, Nuaillé, France), 100 U/ml penicillin and 100 μg/ml streptomycin (Gibco, ThermoFisher Scientific, Waltham, MA, USA). The cells were used for experiments within 20 passages from thawing. LNCaP cells, purchased from American Type Culture Collection (ATCC; CRL-1740) and used within five passages from arrival, were grown in RPMI 1640 Medium (ATCC modification) A10491 (Gibco) supplemented with 10% FBS, 100 units/ml penicillin and 100 μg/ml streptomycin.

### Minigene splicing assay

COS-7 or LNCaP cells were plated in 6-well plates, transfected on day 2 and lysed in TRIsure (Meridian Bioscience, Cincinnati, OH, USA) on day 3. RNA was extracted according to the manufacturer’s instruction with the addition of a chloroform extraction. One microgram RNA was reverse transcribed using oligo (dT) primers and the ProtoScript® II First Strand cDNA Synthesis Kit (New England Biolabs, Ipswich, MA, USA) or the iScript Select cDNA Systems Kit (Bio-Rad Laboratories, Hercules, CA, USA). PCR was performed using the primers dUSD2 and dUSA4 (59). The PCR-products were separated in an agarose gel and photographed, and the band intensities were quantified using the ImageJ 1.53a software and evaluated for statistical difference (*P* < 0.05, two-tailed) using the Wilcoxon rank-sum test. To analyze splice site usage, the PCR-products were TA-cloned using the StrataClone PCR Cloning Kit (Agilent, Santa Clara, CA, USA) and analyzed by colony PCR using T3 and T7 primers followed by restriction enzyme digestion with Tth111I.

### RT-PCR for splice site analysis

One microgram of total RNA isolated as described above was reverse transcribed using oligo(dT) primers and random hexamers and the Maxima H Minus First Strand cDNA Synthesis Kit, with dsDNase (ThermoFisher Scientific), including the dsDNase inactivation step. The reaction was incubated for 10 min at 25°C followed by 30 min at 50°C. The following primers were used in PCR: exon 2 forward 5′-GAGAAGAGGGGCTCTTACGG-3′, intron 3 reverse 5′-TTTGGGTTCAAGCGATTCTC-3′, intron 4 reverse 5′-AACGTGGGAATGAGAAGGTG-3′ and exon 6 reverse 5′-TGGCAGCTTGTTCACTCTGA-3′. The amplified PCR-fragments were excised from the agarose gel, purified and Sanger sequenced.

### Western blot

COS-7 cells plated in 6-well plates were transfected with an ANO7-L expression construct (16), kindly provided by Professor Dr med. Karl Kunzelmann (University of Regensburg, Germany). One and a half day later, the cells were lysed in 200 μl 2X Laemmli Sample Buffer (Bio-Rad Laboratories), and the lysates were sonicated (3 s pulses with 3 s breaks, 50% amplitude) with a Sonopuls ultrasonic homogenizer (Bandelin) until the genomic DNA became fragmented. Finally, the samples were adjusted to 1X Laemmli Sample Buffer concentration containing the indicated amount of β-mercaptoethanol. Five microliter of unheated lysate was separated by 8% SDS-PAGE (sodium dodecyl sulfate polyacrylamide gel electrophoresis), and the proteins were transferred onto a 0.45 μm Immobilon®-P Transfer Membrane (Merck Millipore, Darmstadt, Germany). The membrane was stained with Ponceau S solution (Santa Cruz Biotechnology, Dallas, TX, USA) and scanned using an Epson Perfection V500 Photo Scanner. Blocking was done with 5% weight per volume (w/v) non-fat dry milk in TBST (Tris-buffered saline, TBS, 0.1% v/v Tween 20), incubation with the primary antibody anti-ANO7 HPA035730 (Sigma-Aldrich, Merck, Darmstadt, Germany) at a 1:1000 dilution was in 5% w/v BSA (bovine serum albumin) in TBST overnight at +4°C, and incubation with the secondary antibody Goat Anti-Rabbit IgG H&L (immunoglobulin G heavy and light chain; horseradish peroxidase, HRP) preadsorbed ab97080 (Abcam, Cambridge, UK) in 5% w/v non-fat dry milk in TBST. The secondary antibody was detected using WesternBright™ Quantum western blotting detection kit (Advansta, San Jose, CA, USA) and the membrane was exposed using a Fujifilm LAS-4000 Luminescent Image Analyzer.

### Immunohistochemistry

Tissue sampling and sectioning were performed as described (27). The patient PrCa-34.84 underwent lesion-targeted ablation using magnetic resonance imaging (MRI)-guided transurethral ultrasound, and consequently, post-surgery tissue processing followed a different sampling and fixation protocol (60). For immunohistochemistry, the sections were deparaffinized with xylene and rehydrated. Antigen retrieval was performed for 20 min in a pressure cooker (Decloaking Chamber™ NxGen, Biocare Medical, Pacheco, CA, USA) using citrate buffer pH 6 (Genmed, Cwmbran, UK or Nordic BioSite, Täby, Sweden) followed by washes in 50 mm Tris–HCl pH 7.6, 0.05% v/v Tween 20. The following steps were performed in a Lab Vision Autostainer (ThermoFisher Scientific). The slides were treated with 3% v/v H_2_O_2_ for 10 min, washed, and then incubated with Normal antibody diluent (ImmunoLogic, WellMed, Duiven, The Netherlands) for 10 min. ANO7 was detected with anti-ANO7 HPA035730 diluted 1:200 (Sigma-Aldrich, Merck) for 1 h at room temperature. The primary antibody was detected using BrightVision, two components detection system, peroxidase, goat anti-Mouse/Rabbit IgG HRP (ImmunoLogic, WellMed), which was applied at room temperature for 30 min. The substrate BrightDAB (ImmunoLogic, WellMed) was applied for 10 min at room temperature. The slides were counterstained with Mayer’s hematoxylin, dehydrated, treated with xylene and then mounted in Pertex Mounting Medium. The slides were scanned with a Pannoramic 1000 scanner (3DHISTECH, Budapest, Hungary) equipped with an Adimex Q-12A-180Fc camera and a Plan-Apochromat 20X objective, and images were captured using CaseViewer software version 2.3.

## Supplementary Material

Wahlstrom_Supplementary_Fig1_16-12-2021_ddac012

Wahlstrom_Supplementary_Fig2_15-10-2021_ddac012

Wahlstrom_Supplementary_Table_S1_15-10-2021_ddac012

Wahlstrom_Supplementary_Table_S2_15-10-2021_ddac012

Wahlstrom_Supplementary_Table_S3_15-10-2021_ddac012

Wahlstrom_Supplementary_Table_S4_15-10-2021_ddac012

Wahlstrom_Supplementary_Table_S5_15-10-2021_ddac012

Wahlstrom_Supplementary_Table_S6_16-12-2021_ddac012

Wahlstrom_Supplementary_information_16-12-2021_ddac012
