# Supplementary figures and images for: The variant rs77559646 associated with aggressive prostate cancer disrupts ANO7 mRNA splicing and protein expression
Source: Hum Mol Genet. 2022 Jan 19;31(12):2063–77. doi: 10.1093/hmg/ddac012 (PMC9239746; doi:10.1093/hmg/ddac012)

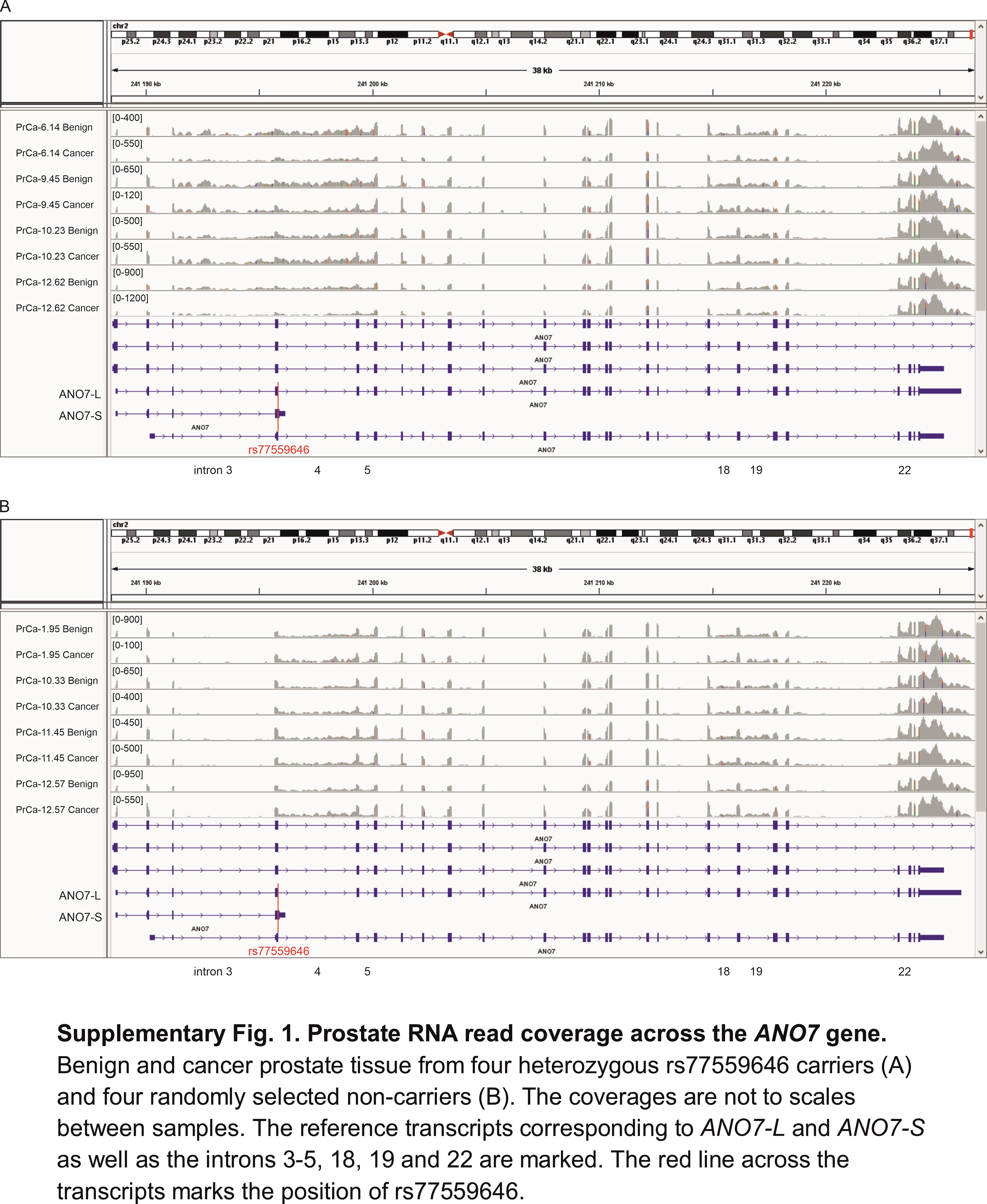

Supplement: Wahlstrom_Supplementary_Fig1_16-12-2021_ddac012 [file wahlstrom_supplementary_fig1_16-12-2021_ddac012.jpeg]

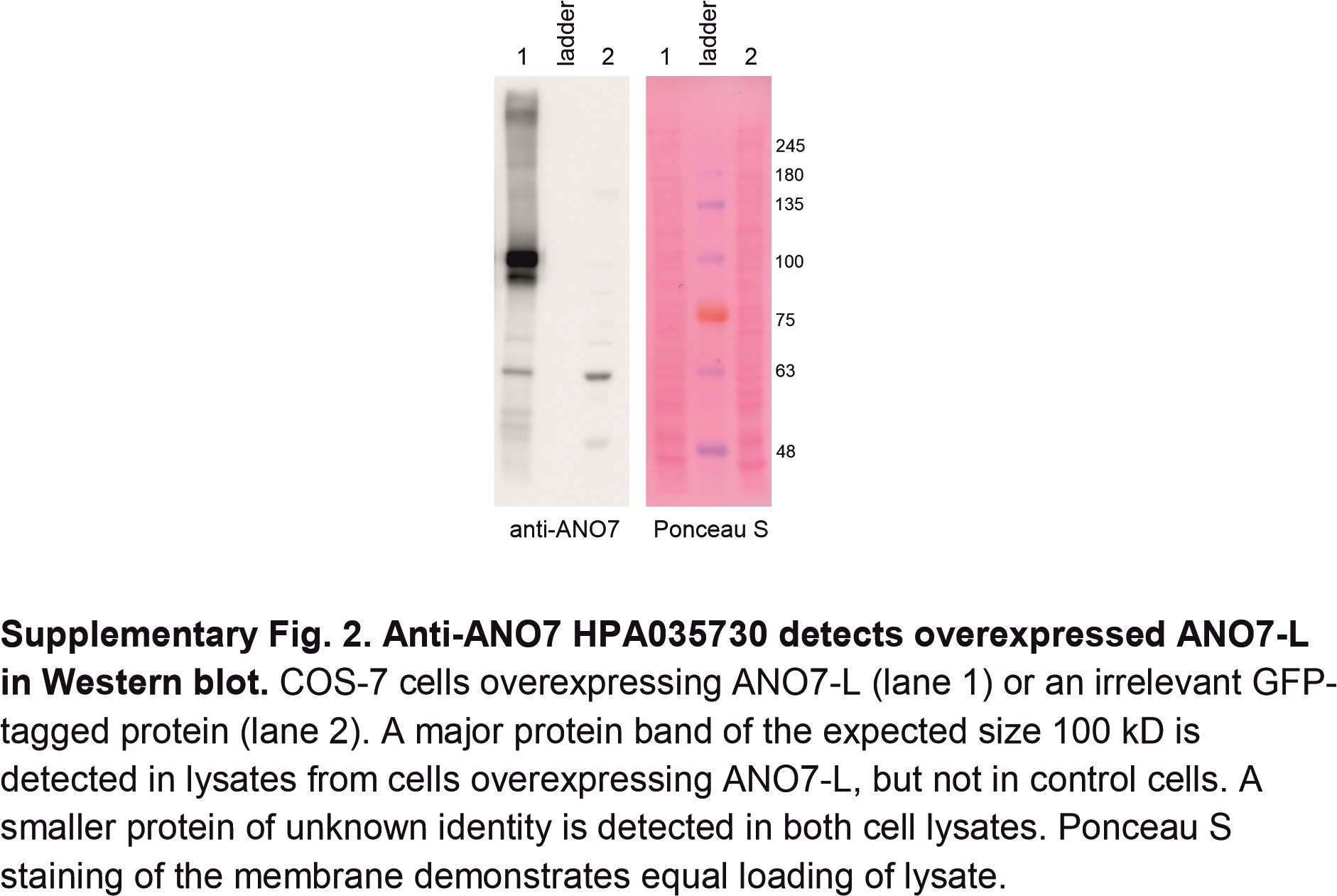

Supplement: Wahlstrom_Supplementary_Fig2_15-10-2021_ddac012 [file wahlstrom_supplementary_fig2_15-10-2021_ddac012.jpeg]
