# Supplementary material for: The variant rs77559646 associated with aggressive prostate cancer disrupts ANO7 mRNA splicing and protein expression
Source: Hum Mol Genet. 2022 Jan 19;31(12):2063–77. doi: 10.1093/hmg/ddac012 (PMC9239746; doi:10.1093/hmg/ddac012)
Supplement: Wahlstrom_Supplementary_information_16-12-2021_ddac012 [file wahlstrom_supplementary_information_16-12-2021_ddac012.doc]

**SUPPLEMENTARY INFORMATION**

Translation across the reported aberrant splicing events reported by IRFinder, rMATS and LeafCutter, starting with Met in exon 2. Reference splicing according to ANO7-L upstream and downstream of the indicated splicing events is assumed. The unique sequence of each peptide is underlined. Nonsense-mediated decay is predicted for transcripts, in which the stop codon resides more than 50 bp upstream of an exon-exon junction.

##

**NCBI Reference Sequence: NP_001357623.1 (ANO7-L)**

MLRRRAQEEDSTVLIDVSPPEAEKRGSYGSTAHASEPGGQQAAACRAGSPAKPRI**A**DFVLVWEEDLKLDRQQDSAARDRTDMHRTWRETFLDNLRAAGLCVDQQDVQDGNTTVHYALLSASWAVLCYYAEDLRLKLPLQELPNQASNWSAGLLAWLGIPNVLLEVVPDVPPEYYSCRFRVNKLPRFLGSDNQDTFFTSTKRHQILFEILAKTPYGHEKKNLLGIHQLLAEGVLSAAFPLHDGPFKTPPEGPQAPRLNQRQVLFQHWARWGKWNKYQPLDHVRRYFGEKVALYFAWLGFYTGWLLPAAVVGTLVFLVGCFLVFSDIPTQELCGSKDSFEMCPLCLDCPFWLLSSACALAQAGRLFDHGGTVFFSLFMALWAVLLLEYWKRKSATLAYRWDCSDYEDTEERPRPQFAASAPMTAPNPITGEDEPYFPERSRARRMLAGSVVIVVMVAVVVMCLVSIILYRAIMAIVVSRSGNTLLAAWASRIASLTGSVVNLVFILILSKIYVSLAHVLTRWEMHRTQTKFEDAFTLKVFIFQFVNFYSSPVYIAFFKGRFVGYPGNYHTLFGVRNEECAAGGCLIELAQELLVIMVGKQVINNMQEVLIPKLKGWWQKFRLRSKKRKAGASAGASQGPWEDDYELVPCEGLFDEYLEMVLQFGFVTIFVAACPLAPLFALLNNWVEIRLDARKFVCEYRRPVAERAQDIGIWFHILAGLTHLAVISNAFLLAFSSDFLPRAYYRWTRAHDLRGFLNFTLARAPSSFAAAHNRTCRYRAFRDDDGHYSQTYWNLLAIRLAFVIVFEHVVFSVGRLLDLLVPDIPESVEIKVKREYYLAKQALAENEVLFGTNGTKDEQPEGSELSSHWTPFTVPKASQLQQ

The amino acid A in bold font is encoded by the first codon of exon 4. The first predicted transmembrane domain (1) is double underlined.

##

**NCBI Reference Sequence: NP_001001666.2 (ANO7-S)**

MLRRRAQEEDSTVLIDVSPPEAEKRGSYGSTAHASEPGGQQAAACRAGSPAKPRIDFVLVWEEDLKLDRQQDSAARDRTDMHRTWRETFLDNLRAAGLCVDQVRGGCHGQGPRPCIHSVTHDLAA

Splicing of ANO7-S utilizes the downstream splice acceptor site of intron 3, resulting in loss of one A at the exon 3 – exon 4 junction. Polyadenylation and cleavage following position 241196165 in intron 4, stop codon at position 241195915. The underlined sequence is encoded by intron 4.

##

**IRFinder, intron 3 retention**

MLRRRAQEEDSTVLIDVSPPEAEKRGSYGSTAHASEPGGQQAAACRAGSPAKPRIGEPLPAPVPHPCWS*

Stop codon at position 241191293 in intron 3.

ANO7-L polyadenylation: Nonsense-mediated decay.

ANO7-S polyadenylation: Modified ANO7 short isoform.

##

**IRFinder, intron 4 retention**

MLRRRAQEEDSTVLIDVSPPEAEKRGSYGSTAHASEPGGQQAAACRAGSPAKPRIADFVLVWEEDLKLDRQQDSAARDRTDMHRTWRETFLDNLRAAGLCVDQVRGGCHGQGPRPCIHSVTHDLAA*

Stop codon as in ANO7-S.

ANO7-L polyadenylation: Nonsense-mediated decay.

ANO7-S polyadenylation: Not applicable.

##

**IRFinder, intron 5 retention**

MLRRRAQEEDSTVLIDVSPPEAEKRGSYGSTAHASEPGGQQAAACRAGSPAKPRIADFVLVWEEDLKLDRQQDSAARDRTDMHRTWRETFLDNLRAAGLCVDQQDVQDGNTTVHYALLSASWAVLCYYAEDLRLKLPLQVRGRHGDRVGLEVPVPTHCVQLPVPTASGGLPAQRPQGLLLPTPTPGPKKPHWANRAGGGIPHSGLPGGGVG*

Stop codon at position 241199640 in intron 5.

ANO7-L polyadenylation: Nonsense-mediated decay.

ANO7-S polyadenylation: Not applicable.

##

**rMATS event ID 12722 (Benign), 12384 (Cancer)**

Skipped exon: Exon 4 reference. Upstream exon: Exon 3 extended in the 5' end with 9 nucleotides. Downstream exon: Exon 5 reference.

MLRRRAQEEDSTVLIDVSPPEAEKRGSYGSTAHASEAFQPGGQQAAACRAGSPAKPRIAGRPGREHHSALRPPQRLLGCALLLRRRPAPEAALAGVTQPGLQLVGRPAGMAGHPQRPAGGCARRTPRVLLLPVQSEQAATLPRE*

Frameshift and stop codon at position 241201310 in exon 7.

ANO7-L polyadenylation: Nonsense-mediated decay.

ANO7-S polyadenylation: Not applicable.

##

**rMATS event ID 12612 (Benign)**

Skipped exon: Cryptic exon in intron 3 (153 bp, 241191763-241191915). Upstream exon: Exon 3 reference. Downstream exon: Exon 4 ANO7-S reference.

MLRRRAQEEDSTVLIDVSPPEAEKRGSYGSTAHASEPGGQQAAACRAGSPAKPRIGWLLLKKNHKC*

Stop codon at position 241191795 in intron 3.

ANO7-L polyadenylation: Not applicable.

ANO7-S polyadenylation: Nonsense-mediated decay.

##

**rMATS event ID 12627 (Benign)**

Skipped exon: Cryptic exon in intron 3 (189 bp, 241194842-241195030). Upstream exon: Exon 3 reference. Downstream exon: Exon 4 truncated in the 5' end with 3 nucleotides.

MLRRRAQEEDSTVLIDVSPPEAEKRGSYGSTAHASEPGGQQAAACRAGSPAKPRIVSGIHRVLG*

Stop codon at position 241194868 in intron 3.

ANO7-L polyadenylation: Nonsense-mediated decay.

ANO7-S polyadenylation: Nonsense-mediated decay.

##

**rMATS event ID 12688 (Benign)**

Skipped exon: Cryptic exon in intron 3 (247 bp, 241191669-241191915). Upstream exon: Exon 3 reference. Downstream exon: Exon 4 truncated in the 5' end with 3 nucleotides.

MLRRRAQEEDSTVLIDVSPPEAEKRGSYGSTAHASEPGGQQAAACRAGSPAKPRIGDCTSTSRAPSTHPAHPAVPSAPERP*

Stop codon at position 241191746 in intron 3.

ANO7-L polyadenylation: Nonsense-mediated decay.

ANO7-S polyadenylation: Nonsense-mediated decay.

##

**rMATS event ID 3021 (Benign), 3044 (Cancer)**

Long exon: Exon 7 extended in the 3' end with 169 nucleotides. Short exon: Exon 7 reference. Flanking exon: Exon 8 reference.

LeafCutter events Intron 7, 169 nt downstream of exon 7 > Exon 8 and Intron 7, 455 nt downstream of exon 7 > Exon 8

MLRRRAQEEDSTVLIDVSPPEAEKRGSYGSTAHASEPGGQQAAACRAGSPAKPRIADFVLVWEEDLKLDRQQDSAARDRTDMHRTWRETFLDNLRAAGLCVDQQDVQDGNTTVHYALLSASWAVLCYYAEDLRLKLPLQELPNQASNWSAGLLAWLGIPNVLLEVVPDVPPEYYSCRFRVNKLPRFLGSDNQDTFFTSTKRHQIVSGGSLPRAPNPDLALRILPAPSLHGCRKACLRPEGRGLEPGGFGNFRG*

Stop codon at position 241201503 in intron 7.

ANO7-L polyadenylation: Nonsense-mediated decay.

ANO7-S polyadenylation: Not applicable.

##

**LeafCutter Cancer event rank a**

Exon 3 > Exon 5

MLRRRAQEEDSTVLIDVSPPEAEKRGSYGSTAHASEPGGQQAAACRAGSPAKPRIAGRPGREHHSALRPPQRLLGCALLLRRRPAPEAALAGVTQPGLQLVGRPAGMAGHPQRPAGGCARRTPRVLLLPVQSEQAATLPRE*

Frameshift and stop codon at position 241201310 in exon 7.

ANO7-L polyadenylation: Nonsense-mediated decay.

ANO7-S polyadenylation: Not applicable.

##

**LeafCutter Cancer event rank d**

Exon 3 > Intron 4 position 241197672, 1827 nt downstream of exon 4

MLRRRAQEEDSTVLIDVSPPEAEKRGSYGSTAHASEPGGQQAAACRAGSPAKPRIDRV*

Stop codon at position 241197680 in intron 4.

ANO7-L polyadenylation: Modified ANO7 long isoform if the 5’ splice site is located upstream of the stop codon and the number of bases in the resulting cryptic exon is a multiple of three. Otherwise nonsense-mediated decay.

ANO7-S polyadenylation: Not applicable.

##

**LeafCutter Cancer event rank e**

Exon 3 > Intron 3 position 241192002, 751 nt downstream of exon 3

MLRRRAQEEDSTVLIDVSPPEAEKRGSYGSTAHASEPGGQQAAACRAGSPAKPRIAGRRAFVCWDCHKKYHSLGQAQWLMPVIPALWEAKAGGSPEVGSSRPA*

Stop codon at position 241192145 in intron 3.

ANO7-L polyadenylation: Modified ANO7 long isoform if the 5’ splice site is located upstream of the stop codon and the number of bases in the resulting cryptic exon is a multiple of three. Otherwise nonsense-mediated decay.

ANO7-S polyadenylation: Modified ANO7 short isoform if the 5’ splice site is located upstream or less than 50 bp downstream of the stop codon. Otherwise nonsense-mediated decay.

##

**LeafCutter Cancer event rank f**

Exon 4 > Internal exon 5 position 241199319, 3 nt missing from the 5' end of the exon

ANO7-L polyadenylation: Missing amino acid Q.

ANO7-S polyadenylation: Not applicable.

##

**LeafCutter Cancer event rank g**

Exon 4 > Internal exon 5 position 241199363, 47 nt missing from the 5' end of the exon

MLRRRAQEEDSTVLIDVSPPEAEKRGSYGSTAHASEPGGQQAAACRAGSPAKPRIADFVLVWEEDLKLDRQQDSAARDRTDMHRTWRETFLDNLRAAGLCVDQRLLGCALLLRRRPAPEAALAGVTQPGLQLVGRPAGMAGHPQRPAGGCARRTPRVLLLPVQSEQAATLPRE*

Frameshift and stop codon at position 241201310 in exon 7.

ANO7-L polyadenylation: Nonsense-mediated decay.

ANO7-S polyadenylation: Not applicable.

##

**LeafCutter Cancer event rank i**

Exon 4 > Intron 4 position 241197672, 1827 nt downstream of exon 4

MLRRRAQEEDSTVLIDVSPPEAEKRGSYGSTAHASEPGGQQAAACRAGSPAKPRIADFVLVWEEDLKLDRQQDSAARDRTDMHRTWRETFLDNLRAAGLCVDQTGSESVTQAGVQWCDLGSLQLLSPGFK*

Stop codon at position 241197753 in intron 4.

ANO7-L polyadenylation: Modified ANO7 long isoform if the 5’ splice site is located upstream of the stop codon and the number of bases in the resulting cryptic exon is a multiple of three. Otherwise nonsense-mediated decay.

ANO7-S polyadenylation: Not applicable.

##

**LeafCutter Cancer event j_1**

Exon 4 > Intron 4 position 241199288, 28 nt upstream of exon 5

MLRRRAQEEDSTVLIDVSPPEAEKRGSYGSTAHASEPGGQQAAACRAGSPAKPRIADFVLVWEEDLKLDRQQDSAARDRTDMHRTWRETFLDNLRAAGLCVDQALTEPWVPTAGRPGREHHSALRPPQRLLGCALLLRRRPAPEAALAGVTQPGLQLVGRPAGMAGHPQRPAGGCARRTPRVLLLPVQSEQAATLPRE*

Frameshift and stop codon at position 241201310 in exon 7.

ANO7-L polyadenylation: Nonsense-mediated decay.

ANO7-S polyadenylation: Not applicable.

##

**LeafCutter Cancer event j_2**

Intron 4 position 241197793, 1948 nt downstream of exon 4 > Exon 5

Translations in three frames from the stop codon preceeding the 3’ end of the cryptic exon:

Frame 1, stop at 241197511:

*VAGTTGPHHHARLIFCRDEVSLCCPGWFGTPGLKQSACLSLPSHLPFFFFFFRQGLNLSPRLEYSGVILAHYNFCLLGSSDSPASASRVAGITX

Frame 2, stop at 241197779:

*LGLQ

Frame 3, stop at 241197753:

*FSCLSLPSSWDYX

ANO7-L polyadenylation: Modified ANO7 long isoform if the 5’ splice site is located downstream of the stop codon in frame 2 and the number of bases in the resulting cryptic exon is a multiple of three. Otherwise nonsense-mediated decay.

ANO7-S polyadenylation: Not applicable.

##

**LeafCutter Cancer event j_3**

Intron 4 position 241197793, 1948 nt downstream of exon 4 > Internal exon 5, 3 nt missing from the 5' end of the exon

Translations in three frames from the stop codon preceeding the 3’ end of the cryptic exon:

Frame 1, stop at 241197511:

*VAGTTGPHHHARLIFCRDEVSLCCPGWFGTPGLKQSACLSLPSHLPFFFFFFRQGLNLSPRLEYSGVILAHYNFCLLGSSDSPASASRVAGITX

Frame 2, stop at 241197779:

*LGLQ

Frame 3, stop at 241197753:

*FSCLSLPSSWDYX

ANO7-L polyadenylation: Modified ANO7 long isoform if the 3’ splice site is located downstream of the stop codon in frame 2 and the number of bases in the resulting cryptic exon is a multiple of three. Otherwise nonsense-mediated decay.

ANO7-S polyadenylation: Not applicable.

##

**LeafCutter Cancer event NA**

Exon 3 > Intron 3 position 241194842, 3591 nt downstream of exon 3

MLRRRAQEEDSTVLIDVSPPEAEKRGSYGSTAHASEPGGQQAAACRAGSPAKPRIVSGIHRVLG*

Stop codon at position 241194868 in intron 3.

ANO7-L polyadenylation: Modified ANO7 long isoform if the 5’ splice site is located upstream of the stop codon and the number of bases in the resulting cryptic exon is a multiple of three. Otherwise nonsense-mediated decay.

ANO7-S polyadenylation: Modified ANO7 short isoform if the 5’ splice site is located upstream or less than 50 bp downstream of the stop codon. Otherwise nonsense-mediated decay.

##

**References**

 1. Das, S., Hahn, Y., Walker, D.A., Nagata, S., Willingham, M.C., Peehl, D.M., Bera, T.K., Lee, B. and Pastan, I. (2008) Topology of NGEP, a prostate-specific cell:cell junction protein widely expressed in many cancers of different grade level. *Cancer Res.*, **68**, 6306–6312.
